# Impact of Nonpharmacological Public Health Interventions on Epidemiological Parameters of COVID-19 Pandemic in India

**DOI:** 10.7759/cureus.15393

**Published:** 2021-06-02

**Authors:** Pradip Kharya, Anil R Koparkar, Anand M Dixit, Hari S Joshi, Rama S Rath

**Affiliations:** 1 Department of Community Medicine & Family Medicine, All India Institute of Medical Sciences, Gorakhpur, Gorakhpur, IND; 2 Department of Community Medicine & Family Medicine, All India Institute of Medical Sciences, Gorakhpur, Gorakhpur, India, IND

**Keywords:** covid-19, india, public health interventions

## Abstract

Background

Public health interventions are epidemiologically sound and cost-effective methods to control disease burden. Non-pharmacological public health interventions are the only mode to control diseases in the absence of medication.

Objective

To find the impact of public health interventions on the epidemiological indicators of disease progression.

Methods

This is a secondary data analysis done on COVID-19 data. The median doubling time and R0 were calculated for a rolling period of seven days. Interventions were scored from zero to three with an increasing level of stringency. Multivariate linear regression was performed to find the role of individual interventions on R0 and the median doubling time.

Results

The highest intervention score was reported in the lockdown phase, which gradually decreased to the lowest level of 22. The R0 values settled to a level of 1.25, and the median doubling time increased to 20 days at the end of the study. Public awareness and public health laws were found to be related to both R0 and the median doubling time in the pre-lockdown phase only.

Conclusion

The implementation of interventions at the ground level is one of the key factors in the success of public health interventions. Post implementation, poor effectiveness of many interventions is evident from the study. Further, studies related to the sequence of interventions are required to further analyze the poor effect of the interventions.

## Introduction

Public health interventions (PHIs) promote or protect good health or prevent ill health in communities or populations [1]. They are the most cost-effective and epidemiologically sound methods to tackle both communicable and non-communicable diseases [2]. From the Spanish flu pandemic in the early twentieth century to the current period, PHIs have played an important role in preventing rapid human massacre [3,4].

The recent COVID-19 pandemic has activated the public health system of every country. Every country has started intervening in the problem in the most innovative public health approaches, apart from clinical approaches. In the absence of appropriate treatment for COVID-19, PHIs remains the only measure to tackle this pandemic. PHIs may include two types of interventions: 1) pharmacological, including vaccines, and other biologicals, and 2) non-pharmacological interventions (NPHIs), like wearing masks, physical distancing, hand washing, and testing and tracing. NPHIs have been significantly helpful in controlling pandemics in the past.

Since its first notification in Wuhan province of China, the COVID-19 pandemic has spread to almost all countries and severely impacted human lives [5]. In India, the pandemic began in late January 2020, approximately one month after the start of the pandemic in the world [6]. The government of India (GOI) accordingly took many steps to stop the progress of the pandemic in India. The steps included imposing a ban on out-migration and immigration from the country, imposing COVID-19-appropriate behaviour, invoking the Epidemic Diseases Act, making the disease notifiable, closing all nonessential services, and, finally, imposing a lockdown. 

The main objective of the study is to assess the nature and type of NPHIs in different phases of the COVID-19 pandemic in India, and their impact on the various epidemiological indicators related to disease progression.

## Materials and methods

Study type

This study was a secondary data analysis conducted on data collected from different sources reporting COVID-19 caseload and intervention.

Study duration

The study was conducted from January 2020 to June 2020. 

Methodology

The required data regarding the COVID-19 pandemic was collected from the Ministry of Health and Family Welfare (MOHFW), Government of India (GOI) and Press Information Bureau of India (PIB). Interventions related to COVID-19 were collected from the Ministry of Home Affairs (MHA), GOI website, MOHFW, and PIB.

Data analysis

Data collected were entered into Microsoft Excel 2019 (Microsoft Corporation, Redmond, USA) and the statistical analysis was done in Microsoft Excel 2019 and Stata 12 (StataCorp, College Station, USA).

*Classification of Time Periods: *The whole period of the epidemic was classified into seven phases according to the interventions and caseload. The detailed method of classification is presented in Table 1. Phase 1 started from the day GOI took the first initiative against the pandemic, i.e., 21 January 2020. 

**Table 1:** Classification of the Study Period PHIs: public health interventions

Phase of Pandemic	Intervention and Caseload Classification	Time Period
Phase-1	Low caseload and less PHIs	21/01/20- 01/03/20
Phase-2	Low caseload and increasing PHIs	02/03/20- 24/03/30
Phase-3	Phase-1 of Lockdown	25/03/20- 14/4/20
Phase-4	Phase-2 of Lockdown	15/4/20- 03/05/20
Phase-5	Phase-3 of Lockdown	04/05/20- 17/05/20
Phase-6	Phase-4 of Lockdown	18/05/20- 31/05/20
Phase-7	Phase-1 of Unlocking	01/06/20- 30/06/20

*Classification of the Interventions: *The NPHI measures related to COVID-19 taken by GOI can be categorized into the following domains: restrictions in workplaces; restriction on industries, agriculture, and construction; restrictions on local transport; restrictions on interstate air transport; restriction on outmigration and immigration, physical distancing, closure of educational institutes, closure of the hospitality sector, restrictions on public gatherings, health system preparedness, public awareness, and public health laws.

*Intervention Scoring: *Each intervention was scored from zero to three based on the strictness with which it was prescribed. A higher score indicates stricter intervention and a lower score indicates lesser intervention. The highest intervention score was 42 and the lowest was 0.

*Analysis: *Various parameters like median doubling time, death rate, recovery rate, and R0 (basic reproduction number) related to disease were calculated. The median intervention score, R0, median doubling time, death rate, and recovery rate were calculated for each period. Multivariate linear regression was performed to find the impact of each intervention on the summary measures of the COVID-19 pandemic, i.e., median doubling time and R0. For this purpose, we divided the whole study period into two categories: 1) the pre-lockdown and lockdown period, and 2) the post-lockdown period. A p-value of less than 0.05 was considered statistically significant. 

From the number of cases, the median doubling time and R0 were calculated using the following formula.

Median doubling time = $\frac{ln(2)}{\frac{C1}{C2}}$ days

Where $C1$ is the number of cases in a given day and $C2$ is the number of cases in the previous day.

R0 =  $1+\frac{I\times ln(2)}{T_{d}}$

Where $I$ is the infectious period and $T_{d}$ is the doubling time [7]. 

R0 and the median doubling time were calculated using a sliding time period of seven days. Thus, R0 was calculated from the caseload of the previous seven days.

Study definitions

The following definitions were used in this study.

1. COVID-19 cases: The laboratory-diagnosed cases as indicated by MOHFW.

2. COVID-19 related deaths: A death due to COVID-19 is defined for surveillance purposes as a death resulting from a clinically compatible illness in a probable or confirmed COVID-19 case, unless there is a clear alternative cause of death that cannot be related to COVID disease (e.g., trauma). There should be no period of complete recovery from COVID-19 between illness and death [8]. 

3. Recovered cases: Recovered cases are the cases declared as recovered according to the GOI guidelines. The definition of "cured" changed from time to time during the COVID-19 pandemic [9, 10].

4. Median doubling time: Time is taken by the number of cases to double.

5. Basic reproduction number (R0): The average number of secondary cases generated by a single primary case [11].

## Results

The pandemic began in India approximately one month after the Wuhan outbreak. The first case was reported on 30 January 2020. The number of cases remained stagnant for one month, i.e., until February 2020. Then it began to rise in the first week of March 2020. The number of COVID-19 cases along with the number of deaths kept rising steadily thereafter.

Epidemiological features of the pandemic

Overall, the pandemic recovery rate increased over time from 10% in early March 2020 to 59.1% at the end of June 2020. However, a sudden rise in the recovered number was observed in the last week of May 2020. The death rate, on the other hand, remained on the lower side, i.e., around 3%. The median doubling time of the pandemic was found to be increasing throughout the study period from 1 day at the start of the pandemic to almost 20 days at the end of June 2020. However, the R0 remained static at around 1.2 till the end of the study period, after an initial rise in R0 was observed in February and March 2020. The phase-wise representation of these summary epidemiological variables is provided in Table 2.

**Table 2:** Outcome Indicators Related to Case Load in Different Phases R0: basic reproduction number; NA: not available

	Phase-1	Phase-2	Phase-3	Phase-4	Phase-5	Phase-6	Phase-7
Time Period	23/01/20- 01/03/20	02/03/20- 24/03/30	25/03/20- 13/4/20	14/4/20 - 03/05/20	04/05/20- 16/05/20	17/05/20- 31/05/20	01/06/20- 30/06/20
Cumulative Cases	3	516	8833	30911	45677	96203	376305
Median Doubling time (in days)	1.4	4.6	5.1	10.4	12.4	13.5	18.6
Number of Zero Growth Days	29	0	0	0	0	0	0
Test Positivity Rate	NA	3.8	5.0	3.5	4.1	6.0	7.9
Recovery Rate (%)	0.00	7.55	9.57	33.26	42.49	57.96	64.57
Death Rate (%)	0.00	1.93	3.48	3.26	3.14	2.76	3.00
R0	4.58	1.96	2.05	1.51	1.41	1.35	1.26
Median Intervention Score	3	15	42	39	37	33	22

Interventions and intervention scores

**Figure 1:**
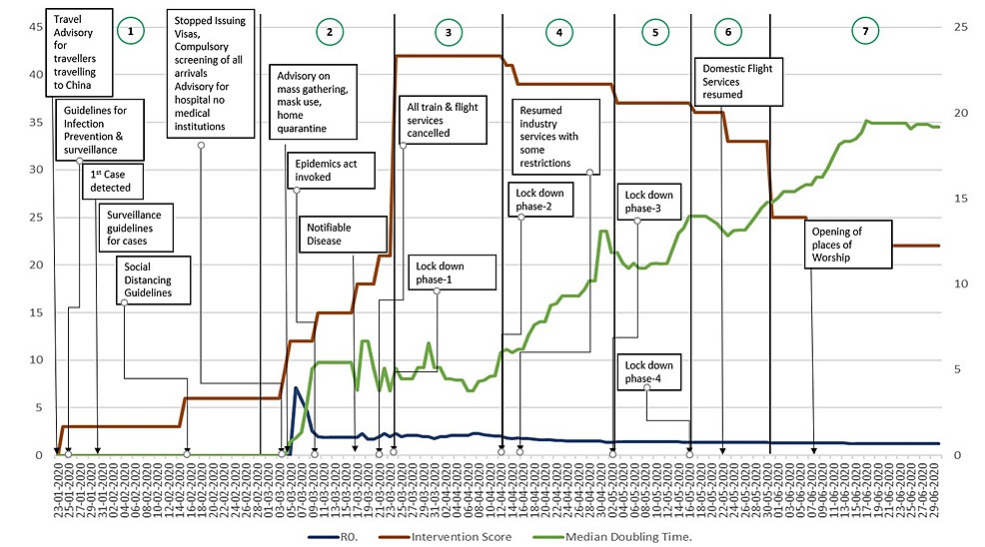
Intervention Score, R0, and Median Doubling Time in the Different Phases of the COVID-19 Pandemic in India R0: basic reproduction rate

India started its preparedness to combat the pandemic before the detection of the first case in India, i.e., on 30 January 2020. The initial preparations were related to outmigration from India, followed by health system preparedness as surveillance. Thus, the initial phases of intervention started from controlling the source of the infection, followed by interventions related to preventing new infections and blocking the transmission. From 25 March 2020, India announced a complete lockdown, followed by a stepwise unlocking of the services. The detailed interventions are provided in Figure 1.

Intervention scores were found to be increasing gradually till 25 March 2020 and attained the highest score of 42. After the aforementioned, there was a gradual decrease in the score in the later phases of lockdown to reach a score of 22 at the end of the study period. The detailed variation of the intervention score is given in Figure 1. The phase-wise variation in the median intervention score is provided in Table 2.

Relation between intervention score and epidemiological features

Through multivariate logistic regression, it was found that public awareness (p<0.05) and public health laws (p<0.05) were significantly associated with the median doubling time in the pre-lockdown phase. Similarly, R0 was negatively associated with public health laws (p<0.05) and positively associated with public awareness (p<0.05) in the pre-lockdown phase. However, immigration, out-migration, and public gathering restrictions and physical distancing were not found to be statistically related to R0 or the median doubling time (Table 3).

**Table 3:** Impact of Interventions on Median Doubling Time and R0 R0: basic reproduction time; R^2^: variance of R0 and the median doubling time explained by the independent variables

	Pre-lockdown				Lockdown & Post-lockdown			
Intervention	Median Doubling Time		R0		Median Doubling Time		R0	
	Coef.	P Value	Coef.	P Value	Coef.	P Value	Coef.	P Value
Restrictions in Offices	NA	NA	NA	NA	-1.3206	>0.05	0.2480	<0.05
Restrictions in Industry / Agriculture	NA	NA	NA	NA	-1.6801	<0.05	0.1214	<0.05
Restrictions in Local Transport	NA	NA	NA	NA	-1.2769	<0.05	0.0666	<0.05
Interstate Air Transport	NA	NA	NA	NA	-0.1897	>0.05	-0.0018	>0.05
Closure of Hospitality	NA	NA	NA	NA	0.4604	>0.05	-0.1097	<0.05
Closure of Educational Institute	NA	NA	NA	NA	Removed due to Collinearity			
Restrictions in Gathering	0.2639	>0.05	-1.35e-^14^	>0.05	Removed due to Collinearity			
Social Distancing	2.41e-16	>0.05	-4.49e-16	>0.05	Removed due to Collinearity			
Closure of Places of Worship	--	--	--	--	-1.0622	<0.05	0.0178	>0.05
Immigration Restrictions	-0.2983	>0.05	0.0846	>0.05	Removed due to Collinearity			
Outmigration Restrictions	0.1196	>0.05	0.0087	>0.05	Removed due to Collinearity			
Public Awareness	1.0486	<0.05	1.4719	<0.05	Removed due to Collinearity			
Public Health Laws	0.6677	<0.05	-0.8403	<0.05	Removed due to Collinearity			
Health System Preparedness	-1.02e-16	>0.05	-6.52e-16	>0.05	Removed due to Collinearity			
R^2^	0.9102		0.8378		0.9477		0.9245	

However, in the lockdown and post-lockdown phase, restriction in industry, agriculture, etc. (p<0.05 ), restrictions in local transport (p<0.05), and closure of places of worship (p<0.05) were found to be negatively associated with the median doubling time, and the same were found to be positively associated with R0. Similarly, closure of the hospitality sector was found to be negatively associated (p<0.05) with R0. The rest of the factors were not found to be significantly associated with the median doubling time and R0 (Table 3).

## Discussion

The study found that proactive intervention started in the early phase of the pandemic in the country. Initially, the interventions were linked to controlling the source of infection, and gradually, they shifted to blocking the transmission and preventing new infections. The median doubling time gradually increased from 1.4 days in Phase 1 to 18.6 days in Phase 7. Similarly, the recovery rate increased gradually to 64.57% in Phase 7. However, R0 decreased gradually after initial fluctuations and settled to a lower level of around 1.2. The death rate, however, remained at a lower level throughout the study period, at around 3%. The intervention scores gradually increased till the end of March 2020, after which a decreasing trend was observed.

The initial interventions were limited to preventing the entry of the infection into the country, which was logically correct as the epicentre of the pandemic was outside India. That was followed by interventions related to the prevention of new infections and blocking the transmission. In the latter part of March 2020, the interventions became multifaceted with the involvement of all sectors. A study by Pan et al. in Wuhan, China showed that the sequence of interventions in China was mainly related to controlling the source of the infection and blocking the transmission routes, followed by the prevention of new infections [12]. In Singapore, the initial interventions were related to the screening of the cases, testing, tracing, and treating the cases, followed by restrictions on flights from other countries [13]. Sri Lanka, which had also reported very few cases till that time, also acted in a similar way [14]. Both the countries shared similar interventions early in the period. 

This study showed that the R0 values gradually decreased in the seven phases of the pandemic in India. This decreasing trend of R0 may be due to the initial availability of a large number of susceptible individuals, which gradually decreased with time to settle the R0 value to a lower level. Interventions linked to the decreasing the contact of infected individuals with suspectable individuals might have resulted in a decrease in R0. Similarly, such trend of R0 values was observed in Severe Acute Respiratory Syndrome (SARS) and Middle East Respiratory Syndrome (MERS) in the past [15]. In the current pandemic also, a study conducted by Chong YC et al. found that the trend of R0 decreased over time with a few peaks in between [16]. A study by You C et al. found that the trend of R0 decreased over time in different states of China [17]. However, the study by Najafi et al. found increasing R0 values after a period of decreasing trend [18]. This increasing trend may be due to decreased stringency of the interventions in the selected province of Iran.

The median doubling time in this study was found to be increasing throughout the study period, with fluctuations in Phase 2 and 3. This increase in median doubling time was noted after the end of Phase 3, which had the highest stringency score in the entire study period. The delay in the rise in doubling time may be due to the time lag between the intervention and getting a visible result on the epidemiological parameters. A study by Muniz-Rodriguez et al. showed a fluctuating nature of the median doubling time when compared with other studies [19]. This fluctuation may be due to the initial phase of the pandemic. A study by Zhou et al. found that the doubling time of countries like Thailand, Australia, Malaysia, Vietnam decreased with time, whereas in countries like Italy, Belarus, and the Philippines, the median doubling time increased over time [20]. This variation may be due to the nature and type of testing and treating strategy adopted by the countries. 

The interventions done by GOI reached maximum stringency level in the latter part of March 2020, i.e., in the third phase of the study. Despite the interventions, the number of cases began to rise in Phase 4 and later in the study period. This may be related to the poor stringency of the interventions or may be due to poor screening of the cases that may have lead to continued transmission in the background. In the pre-lockdown phase, the relation between public awareness and public health laws was found to be significantly associated with the median doubling time. Public awareness was found to be positively associated with R0. This may be because the implementation of public awareness activity might not have transformed into action immediately. However, implementation of the public health laws was found to be negatively associated with the R0, i.e., with strict implementation of public health laws, the R0 value decreased. No statistically significant relation was observed between the factors like physical distancing, immigration, outmigration and R0 and median doubling time. This may be attributed to the fact that the short period of action of these interventions in the pre-lockdown phase. In the lockdown and post-lockdown period, the interventions like the restrictions on industry/agriculture and construction, and restrictions on local transport were found to be negatively associated with the median doubling time and positively associated with R0. A study by Pan et al. showed that the R0 value decreased with interventions [12]. Various modelling studies have also shown the impact of interventions on R0. Studies by Davies et al. and Chowdhury et al. have found that each individual non-pharmacological intervention had some impact on the decrease in R0 [21, 22]. Similar results were obtained by Lai S et al. in their study in China [23]. This opposite relation could be because the closure of workplaces forced many migrant workers to move to their hometowns, which might have led to the seeding of the COVID-19 in the general public [24]. The impact of this was observed in terms of an increase in the caseloads of the states that contribute the largest number of migrant workers [25]. Other interventions also remained nonsignificant in the lockdown and the post-lockdown phase, maybe because of the higher impact of the above-discussed variables.

The study is limited by the fact that the ground-level stringency was not assessed during the study. Similarly, the interventions in the latter phases might have varied depending upon the number of containment zones in each state. This is one of the few studies that have analysed the impact of the various interventions in different phases of the pandemic in the country that accounts for the second-highest number of COVID-19 cases in the world.

## Conclusions

Although strict interventions were planned in India, proper execution of the interventions may have been a problem across the nation, which might have helped the spread of the COVID-19 pandemic in the country. Various interventions that have proven to be helpful in controlling the disease in different parts of the world were found to be ineffective in India. Thus, further research is required in the area of intervention implementation strategies in epidemic situations. Human behavioural patterns in a pandemic situation may be further analyzed to understand the poor effectiveness of interventions as depicted by the current study.
